# Effect of piceatannol-rich passion fruit seed extract on human glyoxalase I–mediated cancer cell growth

**DOI:** 10.1016/j.bbrep.2019.100684

**Published:** 2019-08-30

**Authors:** Takayuki Yamamoto, Akira Sato, Yusuke Takai, Atsushi Yoshimori, Masahiro Umehara, Yoko Ogino, Mana Inada, Nami Shimada, Aya Nishida, Risa Ichida, Ryoko Takasawa, Hiroko Maruki-Uchida, Sadao Mori, Masahiko Sai, Minoru Morita, Sei-ichi Tanuma

**Affiliations:** a Research and Development Institute, Health Science Research Center, Morinaga and Company Limited, 2-1-1 Shimosueyoshi, Tsurumi-ku, Yokohama, 230-8504, Japan; b Faculty of Pharmaceutical Sciences, Tokyo University of Science, 2641 Yamazaki, Noda, Chiba, 278-8510, Japan; c Institute for Theoretical Medicine Inc., 26-1, Muraoka-Higashi 2-chome, Fujisawa, Kanagawa, 251-0012, Japan; d Research Institute for Science and Technology, Organization for Research Advancement, Tokyo University of Science, 2641 Yamazaki, Noda, Chiba, 278-8510, Japan

**Keywords:** Passion fruit seed extract, Piceatannol, Glyoxalase I, Anticancer, PFSE, Passion fruit seed extract, GLO I, glyoxalase I, MG, methylglyoxal, PI3K, phosphoinositide 3-kinase, mTOR, mammalian target of rapamycin, IL-6, interleukin 6, STAT3, signal transducers and activators of transcription 3, MAPK, mitogen-activated protein kinase, HPLC, high-performance liquid chromatography, TCA, tricarboxylic acid

## Abstract

Passion fruit seed extract (PFSE), a product rich in stilbenes such as piceatannol and scirpusin B, has various physiological effects. It is unclear whether PFSE and its stilbene derivatives inhibit cancer cell proliferation via human glyoxalase I (GLO I), the rate-limiting enzyme for detoxification of methylglyoxal. We examined the anticancer effects of PFSE in two types of human cancer cell lines with different GLO I expression levels, NCI–H522 cells (highly-expressed GLO I) and HCT116 cells (lowly-expressed GLO I). PFSE and its stilbenes inhibited GLO I activity. In addition, PFSE and its stilbenes supressed the cancer cell proliferation of NCI–H522 cells more than HCT116 cells. These observations suggest that PFSE can provide a novel anticancer strategy for prevention and treatment.

## Highlights

• Piceatannol, and scirpusin B inhibited GLO I activity.
• Passion fruit seed extract suppressed proliferation and colony formation of NCI–H522 cells.
• Passion fruit seed extract and piceatannol could exert anticancer activity via GLO I inhibition.

## 1 Introduction

Cancer is one of the primary causes of death worldwide; its incidence is increasing. Furthermore, whereas colorectal cancer is the major cause of cancer death in developed countries, lung cancer is the leading cause of cancer death among males in all countries [1]. Numerous anticancer drugs have been developed, but some have side effects such as neurotoxicity, renal toxicity, and bone marrow suppression [2]. Thus, sustained mild suppression of tumors by food constituents such as flavonoids is desired [3,4].

Methylglyoxal (MG) is a byproduct of tumor-specific aerobic glycolysis [5]. It is highly reactive with proteins and DNA/RNA, and is believed to induce apoptosis in tumor cells [6]. MG is detoxified by the rate-limiting enzyme glyoxalase I (GLO I). Many human tumors, including those of the colon and lungs, reportedly have increased GLO I activity [7,8]. For that reason, the GLO I enzyme is believed to be a potential therapeutic target for inducing cancer cell apoptosis. It has been reported that phenylpropanoids such as natural polyphenols represented by flavonoids have GLO I inhibitory activity [9]. For example, quercetin and myricetin have remarkable inhibitory effects due to their high affinity to the binding site of GLO I [10,11]. However, the GLO I inhibitory activity of compounds having a stilbene structure is hardly known.

Piceatannol, a structurally related analog of resveratrol, is a naturally occurring stilbene derivative present at high concentrations in passion fruit (*Passiflora edulis*) seeds. We previously reported that piceatannol displays a variety of biological effects, such as skin protection [12,13], vasodilatation [14], Sirt1 induction [15], and improvement in metabolic health [[16], [17], [18]]. Piceatannol is also a promising natural component for cancer prevention [[19], [20], [21]], because it alters various cellular targets and is antitumorigenic in cell line and animal models [[22], [23], [24]]. The antitumor mechanism of piceatannol involves various pathways, including the phosphoinositide 3-kinase /protein kinase B /mammalian target of rapamycin (PI3K/AKT1/mTOR) [25], spleen tyrosine kinase [26], cyclooxygenase-2 [27], and interleukin 6 /signal transducers and activators of transcription 3 (IL-6/STAT3) pathways [28]. Moreover, piceatannol increases intracellular Ca^2+^ concentrations, activates p38 mitogen-activated protein kinase (MAPK), inactivates extracellular signal–regulated kinase (ERK), and degrades procaspase-8 in leukemic cells [29]. Although Takasawa et al. found that piceatannol inhibits GLO I by binding its active site [30], little is known about the inhibitory effect of passion fruit seed extract (PFSE) and piceatannol on human GLO I–mediated proliferation of cancer cells.

We previously isolated the second major polyphenolic compound in passion fruit seeds: scirpusin B [31]. Scirpusin B is a dimer of piceatannol that was first extracted from Scirpus fluviatilis (Torr.) A. Gray in 1978 by Nakajima et al. [32]. Reports concerning the physiological role of scirpusin B are few, but it has been found to possess anti–human immunodeficiency virus activity [33], α-amylase inhibitory activity [34], and superoxide anion scavenging activity [35]. The anticancer effect of scirpusin B is not known.

In the present study, we investigated the inhibitory activity of PFSE, piceatannol, and scirpusin B against human GLO I. We also examined the antiproliferative effect of PFSE derivatives against cancer cell lines that differed in GLO I expression levels.

## 2 Materials and methods

### 2.1 Chemicals

Piceatannol (>98.0%), resveratrol (>99.0%), rhapontigenin (>98.0%), isorhapontigenin (>96.0%), piceid (>95.0%), pinostilbene (>97.0%), caffeic acid (>98.0%), and *p*-coumaric acid (>98.0%) were obtained from Tokyo Chemical Industry (Tokyo, Japan). Gnetin C (>97.0%), and special grade ethanol (>99.5%) were obtained from FUJIFILM Wako Pure Chemical Corporation (Osaka, Japan). Astringin was obtained from Sequoia Research Products limited company (Pangbourne, United Kingdom). Epicatechin (>90.0%) was obtained from Sigma-Aldrich Japan (Tokyo, Japan). LC-MS grade acetonitrile (>99.9%), LC-MS grade distilled water, and HPLC grade formic acid (>98.0%) were obtained from Kanto Chemical Co., Inc. (Tokyo, Japan).

### 2.2 Preparation of PFSE

Passion fruit seeds were freeze-dried, milled, and extracted with 35% ethanol. After centrifugation, the supernatant was evaporated, and solvent was removed from the pellet by freeze-drying. The lyophilized powder was analyzed by high-performance liquid chromatography (HPLC) as previously described [36].

### 2.3 Materials

Scirpusin B was extracted from passion fruit seeds and purified by HPLC [31]. In brief, the extracts of passion fruit seeds were fractionated by reverse-phase HPLC. Each fraction was collected by an Inertsil ODS-3 column (GL Sciences Inc., Tokyo, Japan) with an (A) water and/or (B) acetonitrile mobile phase at a flow rate of 5 mL/min. A gradient elution of 0–80% (B) at 0–90 min was used for fractionation. The fractionated samples were analyzed by using an ODS-3 column. Analytical HPLC was carried out with an (A) water and/or (B) acetonitrile mobile phase at a flow rate of 0.75 mL/min. A gradient elution of 0–45% (B) at 0–25 min was used for this analysis.

Fetal bovine serum was obtained from Biosera (Kansas City, MO, USA). Roswell Park Memorial Institute (RPMI) 1640 medium and high glucose Dulbecco’s modified Eagle’s medium (DMEM) were obtained from Wako Pure Chemical Industries, Ltd. (Osaka, Japan). Antibiotics were obtained from Wako. All other reagents were obtained from Wako.

### 2.4 Multiple reaction monitoring (MRM) analysis

The purchased standards, and PFSE was analyzed using a Shimadzu Prominence Ultrafast Liquid Chromatograph (UFLC) system equipped with a Kinetex 2.6 μm C18 100 Å ODS column (100 × 2.1 mm i.d.; Phenomenex, Torrance, CA, USA). Elution was carried out at 40 °C using a 0.1% (v/v) formic acid/water solution as mobile phase A, and a 0.1% (v/v) formic acid/acetonitrile solution as mobile phase B. A flow rate of 0.2 mL/min was employed, and the gradient conditions were as follows: 0–5 min, 5% B; 5–25 min, 5–60% B; 25–26 min, 60–95% B; and 26–31 min, 95% B, followed by an isocratic plateau for 7 min and return to the initial conditions (i.e., 5% B). Electrospray ionization tandem mass spectrometry was performed in negative polarity mode using the following settings: Curtain gas, 40 psi; nebulizer gas, 50 psi; turbo gas, 80 psi; capillary temperature, 600 °C; ion spray voltage, −4.5 kV; declustering potential, −20 V; collision energy, −35 to −20 V. The instrument was used in the tandem mass and negative multiple reaction monitoring (MRM) mode.

### 2.5 In vitro GLO I assay

The GLO I assay was performed using a spectrophotometric method for monitoring the increase in absorbance at 240 nm that is induced by formation of *S*-d-lactoylglutathione over a 5 min period at 25 °C [37]. The standard assay mixture contained 7.9 mM MG, 1 mM glutathione, 14.6 mM magnesium sulfate, and 182 mM imidazole-HCl at pH 7.0. The assay mixture was allowed to stand for 15 min to ensure the equilibration of hemithioacetal formation, then recombinant GLO I was added to initiate the reaction.

### 2.6 Molecular docking

The biding modes of scirpusin B and piceatannol were determined using Autodock Vina [38] as molecular docking software. The 3D structures of scirpusin B and piceatannol were prepared by OpenBabel. [39]. A number of crystal structures of human GLO I with its inhibitor were deposited to the Protein Data Bank (PDB) [40]. To date, no crystal structure of human GLO I complexed with flavonoid derivative is available. However, crystal structure of mouse GLO I complexed with baicalein, which is one of the flavonoids, has been published (PDB id: 4×2A) [9]. Therefore, we constructed the homology model of human GLO I based on crystal structure of the mouse GLO I-baicalein complex using SwissModel server [41]. The docking compatible structure formats of the homology model was prepared by AutoDockTools-1.5.7. [42]. For docking with Autodock Vina, the grid size was set to (x, y, z) = (20, 20, 20) and the grid center was set to (x, y, z) = (−10.407, −5.554, 0.829). The other parameters were using the default values.

### 2.7 Cell culture and treatment

Human non–small cell lung cancer NCI–H522 cells and human colon cancer HCT116 cells were purchased from the American Type Culture Collection. NCI–H522 and HCT116 cells were cultured in RPMI 1640 medium and high glucose DMEM supplied with 10% heat-inactivated fetal bovine serum and 100 U penicillin /100 μg/mL streptomycin. The cells were cultured under 5% CO_2_ at 37 °C in a humidified incubator as previously described [43].

### 2.8 Western blot analysis

Western blot analysis was performed as previously described [43,44]. NCI–H522 cells and HCT116 cells were washed in ice-cold phosphate-buffered saline, and then whole cell lysates were prepared using Laemmli sample buffer. Whole cell lysates (5 × 10^4^ cells per lane) were separated by 18% sodium dodecyl sulfate–polyacrylamide gel electrophoresis (SDS-PAGE), and blotted onto a polyvinylidene difluoride membrane (Bio-Rad, Hercules, CA, USA). The membrane was then blocked against nonspecific binding by treatment for 1 h with 5% skim milk in Tris-buffered saline (pH 7.6) that contained 0.1% Tween 20. Then, it was immunoblotted overnight at 4 °C using the respective primary antibodies. Afterward, the membrane was incubated for 1 h at room temperature with horseradish peroxidase-conjugated anti-rabbit immunoglobulin G (IgG) secondary antibodies. The protein bands were visualized using Immobilon Western Chemiluminescent HRP Substrate (Merck Millipore, MA, USA). Protein expression was quantified with a ChemiDoc MP Imaging System (Bio-Rad) using the following antibodies: anti-GLO I antibodies (1:1000; Novus Biologicals, CO, USA), anti-β-actin antibodies (1:20 000; Sigma-Aldrich, St Louis, MO, USA), and anti-rabbit IgG horseradish peroxidase–linked whole antibodies (1:20 000; GE Healthcare, Little Chalfont, Buckinghamshire, England).

### 2.9 WST-8 assay

NCI–H522 cells and HCT116 cells were seeded on 96-well plates at a density of 1000 cells per well. The treated cells were cultured for 24, 48, and 72 h in culture media that included piceatannol, scirpusin B, or PFSE. Then, WST-8 reagent (Dojindo Laboratories, Japan) was added to each well and the cells were incubated for 1 h at 37 °C. Absorbance was measured with a microplate reader at 450 nm.

### 2.10 Colony formation assay

A colony formation assay was performed as previously described [35]. NCI–H522 cells and HCT116 cells were seeded on 6-well plates at a density of 200 cells per well. The treated cells were cultured for 10 d in culture media that included piceatannol, scirpusin B, or PFSE. The cells were fixed with 4% formaldehyde solution and stained with 0.1% (w/v) crystal violet. The visible colonies were counted manually.

### 2.11 Statistical analysis

Data are presented as the mean ± the standard deviation (SD). Statistical analyses (i.e., one-way analysis of variance followed by the Student's *t*-test) were performed using SPSS software (IBM, Armonk, USA.). A *p*-value < 0.05 was considered significant.

## 3 Results

### 3.1 Analysis of PFSE

The analysis of PFSE showed that the PFSE powder contained 104.5 μg/mg piceatannol as the primary polyphenol, as well as 45.5 μg/mg scirpusin B. These two stilbenes were the main compounds of PFSE, and the other compounds were contained in a very small amount as shown in Table 1. In this study, piceatannol and scirpusin B were focused to investigate as well as PFSE.

**Table 1:** Content of each polyphenol in PFSE (MRM mode).

No.	sample	*t*_R_(min)	MRM transition(precursor ion (*m*/*z*) /fragment ion (*m*/*z*))	Collisionenergy (V)	mg/g (in PFSE)
1	Caffeic acid	11.2	179/135	−20	0.00082
2	Epicatechin	12.3	289/245	−35	0.99
3	Astringin	13.1	405/243	−20	0.0068
4	*p*-Coumaric acid	13.5	163/119	−20	0.0023
5	Piceid	14.5	389/227	−20	0.00070
6	Resveratrol	17.1	227/143	−35	0.082
7	Isorhapontigenin	17.7	257/241	−20	0.013
8	Rhapontigenin	18.1	257/241	−20	n.d.
9	Pinostilbene	21.0	241/225	−20	0.00014 ≧
10	Gnetin C	21.1	453/333	−35	n.d.

∗n.d. ・・・not detected.

### 3.2 Effect of PFSE derivatives on human GLO I activity

**Fig. 1:**
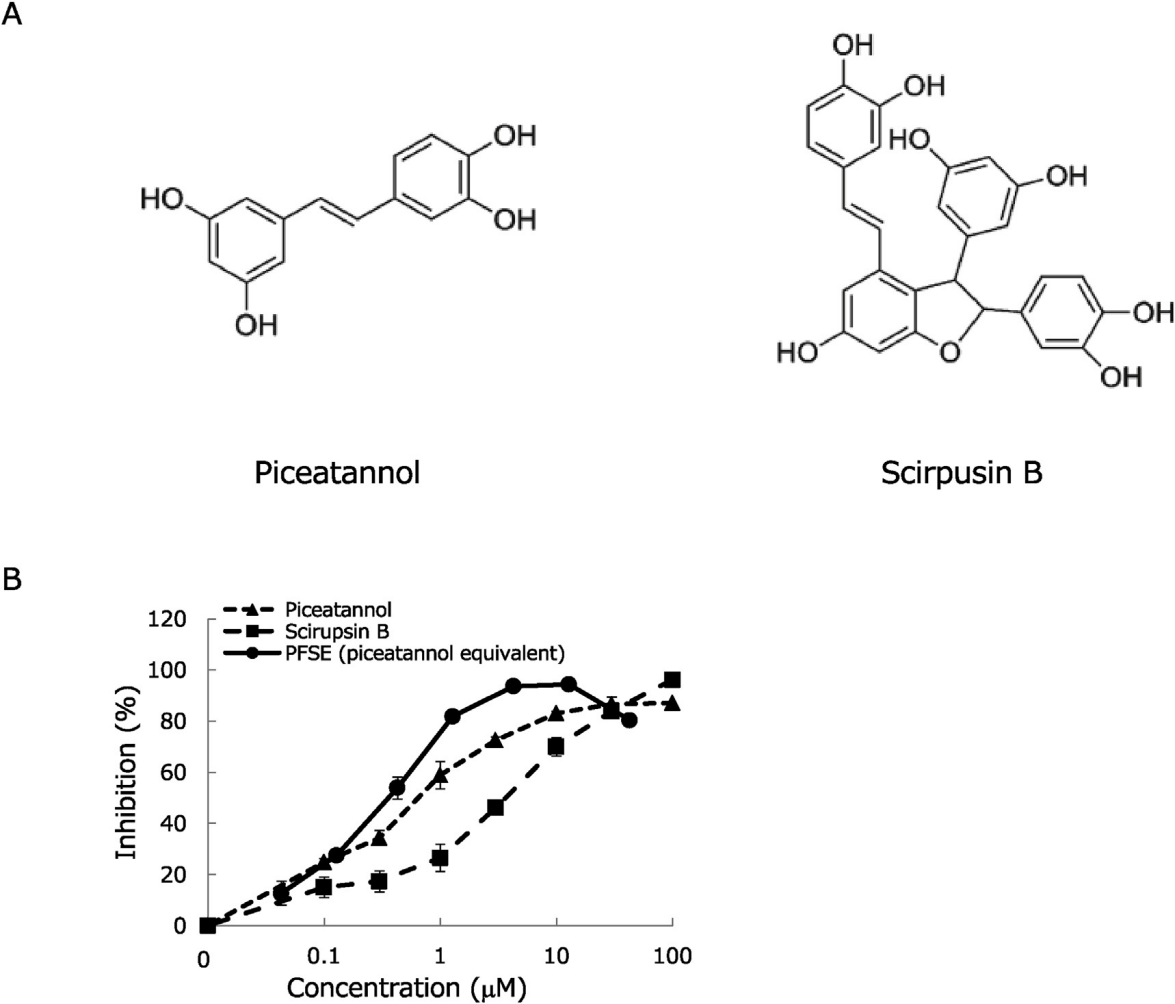
The chemical structures of PFSE derivatives: piceatannol and scirpusin B (A). Inhibitory effects of PFSE, piceatannol, and scirpusin B against human GLO I activity (B). The dose-dependent curves of PFSE, piceatannol, and scirpusin B were measured by an *in vitro* GLO I assay. The values are expressed as the mean ± the standard deviation from 3 independent experiments.

To investigate whether PSFE and its stilbene derivatives (Fig. 1A) inhibit human GLO I, an *in vitro* assay was performed. We evaluated dose dependencies and determined the half maximal inhibitory concentration (IC_50_) values of the extract and derivatives. Results showed that PFSE, piceatannol, and scirpusin B inhibited GLO I in a dose-dependent manner (Fig. 1B). Moreover, the IC_50_ values of piceatannol and scirpusin B were calculated to be 0.75 μM, and 4.2 μM, respectively; and the IC_50_ of the piceatannol equivalent of PFSE was 0.38 μM. These findings suggest that piceatannol and scirpusin B are the primary contributors to PFSE's inhibitory effect on GLO I.

### 3.3 Molecular docking

**Fig. 2:**
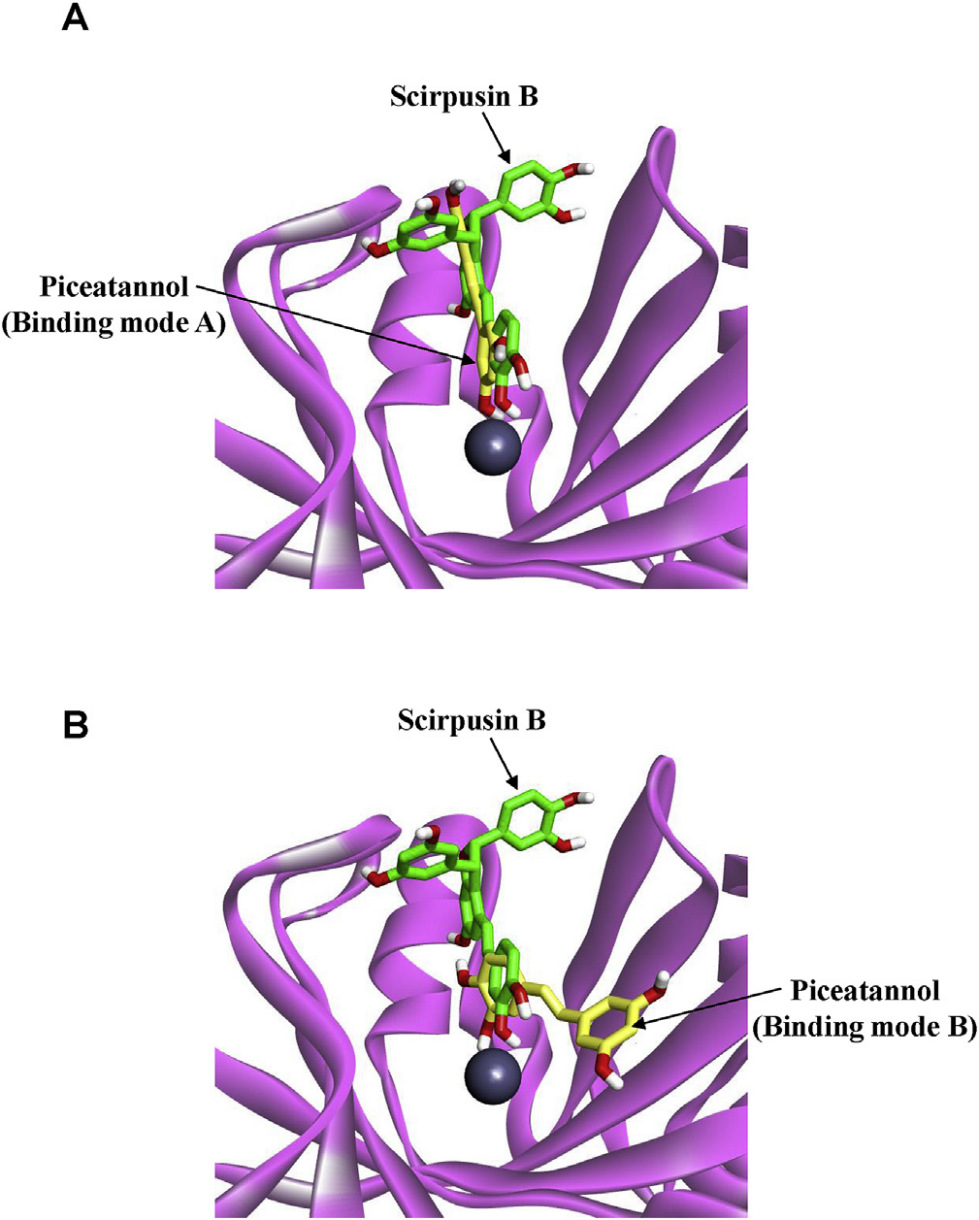
The predicted binding modes of piceatannol or scirpusin B against human GLO I. The ribbons (pink) represent the active site of human GLO I. Carbon and oxygen atoms of piceatannol or scirpusin B are illustrated in green and red sticks. Binding mode A: the predicted binding mode of piceatannol similar to that of baicalein (yellow stick). (A) The predicted binding modes of piceatannol/human GLO I. (B) The predicted binding modes of scirpusin B/human GLO I.

Previously, we reported the binding mode of piceatannol and human GLO I by computational simulation analyses [30]. To understand the predicted binding mode of human GLO I with piceatannol or scrupsin B, we performed docking simulations between the pharmacophore of human GLO I and these compounds, piceatannol or scrupsin B by using the co-crystal structure (PDB: 4×2A) of mouse GLO I/baicalein complex (Fig. 2A and B).

### 3.4 GLO I protein expression levels in NCI–H522 and HCT116 cells

**Fig. 3:**
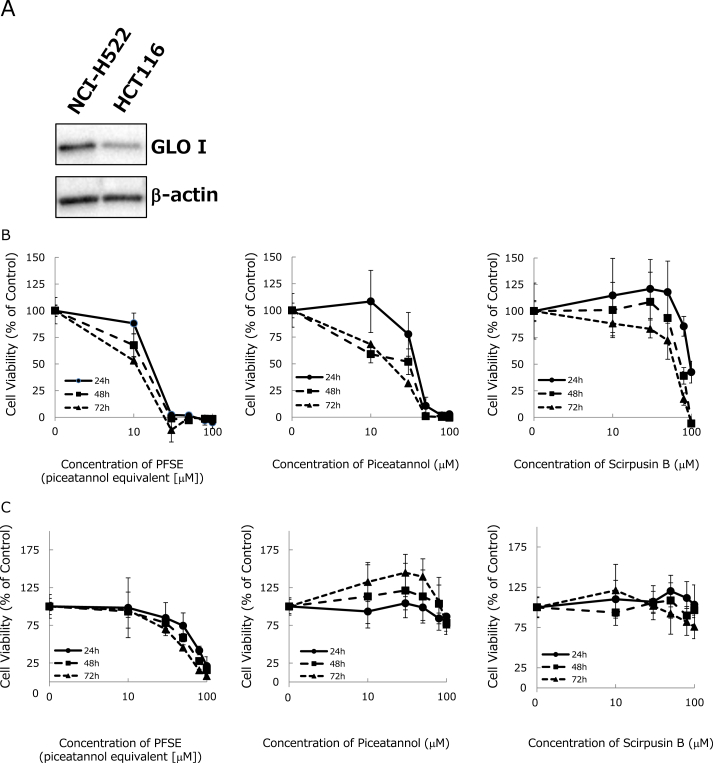
The protein levels of GLO I in NCI–H522 and HCT116 cells. The expression levels of GLO I protein were analyzed by western blotting as described in Section 2.8 (A). The antiproliferative effects of PFSE, piceatannol, and scirpusin B against NCI–H522 cells (B) and HCT116 cells (C). NCI–H522 and HCT116 cells were treated with the indicated concentrations of PFSE, piceatannol, and scirpusin B for 24, 48, and 72 h. The cell viabilities (% of control) were measured by using a WST-8 assay. The values are expressed as the mean ± the standard deviation.

To investigate whether the inhibitory effect of PFSE and stilbenes against GLO I is dependent on enzyme expression levels, two types of cells (*i.e*., NCI–H522 and HCT116) were prepared. Although GLO I gene expression levels for those cell lines have been reported by CellMiner™ (https://discover.nci.nih.gov/cellminer/analysis.do), few reports directly compare the GLO I protein expression levels of NCI–H522 to that of HCT116. Western blot analysis revealed that GLO I protein expression levels were higher in NCI–H522 cells than in HCT116 cells (Fig. 3A). By using cells with different GLO I protein expression levels, the effect of PFSE and stilbenes on cell proliferation could be examined.

### 3.5 Effect of PFSE and stilbenes on cell proliferation

To investigate whether PFSE and stilbenes are effective at suppressing the growth of cells that have different GLO I expression levels, we evaluated the antiproliferative effects of PFSE, piceatannol, and scirpusin B on NCI–H522 cells and HCT116 cells. First, NCI–H522 cells and HCT116 cells were treated with 10, 30, 50, 80, and 100 μM of piceatannol, scirpusin B, or the piceatannol equivalent concentration of PFSE for 24, 48, and 72 h. Cell viabilities (% of control) were measured using the WST-8 assay. Results showed that PFSE, piceatannol, and scirpusin B treatments suppressed proliferation of NCI–H522 cells in a dose- and time-dependent manner (Fig. 3B). In contrast, PSFE had a weak antiproliferative effect against HCT116 cells; moreover, piceatannol and scirpusin B did not suppress HCT116 proliferation (Fig. 3C). The EC_50_ values of PFSE and stilbenes for each cell at 24, 48, and 72 h of treatment were calculated and are shown in Table 2.

**Table 2:** EC_50_ (μM) values as determined by the WST-8 assay.

	NCI–H522			HCT116		
	24 h	48 h	72 h	24 h	48 h	72 h
PFSE	21.1	18.2	15.5	72.3	58.3	46.2
Piceatannol	38.2	30.7	20	>100	>100	>100
Scirpusin B	96.6	74	62.2	>100	>100	>100

EC_50_, half maximal inhibitory concentration. PFSE, passion fruit seed extract.

**Fig. 4:**
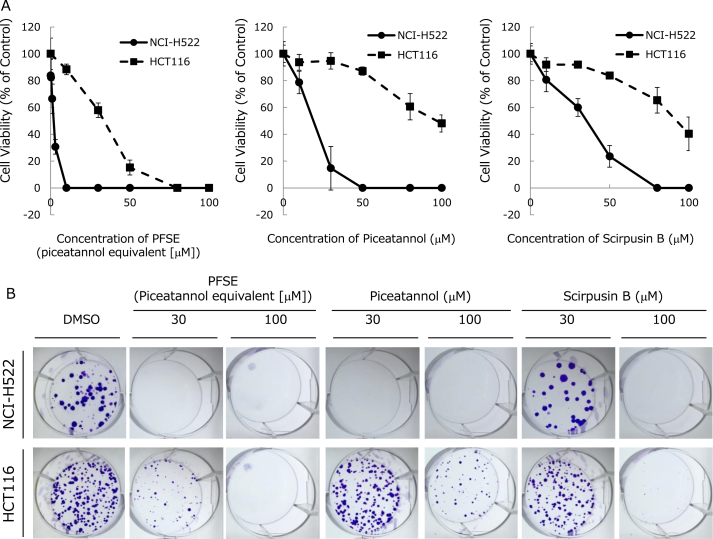
The anticancer effects of PFSE, piceatannol, and scirpusin B in NCI–H522 cells and HCT116 cells. Anticancer activity was assessed by a colony formation test. Cells were treated with the indicated concentrations of PFSE, piceatannol, and scirpusin B for 10 days. (A) Inhibition of colony formation curves, and (B) images of typical colony formation in a 6-well plate after treatment with PFSE, piceatannol, and scirpusin B.

Next, the effects of PFSE and stilbenes on colony formation were investigated. The inhibition curves of colony formation are shown in Fig. 4A, and images of typical colony formation are shown in Fig. 4B. Results of the colony formation experiment correlated with those of the WST-8 assay. That is, PFSE, piceatannol, and scirpusin B efficiently inhibited colony formation in NCI–H522 cells. The EC_50_ values of PFSE, piceatannol, and scirpusin B for NCI–H522 were calculated to be 1.9, 19.0, and 35.5 μM, respectively (Table 3). The inhibitory effect of PFSE and stilbenes against colony formation was greater in NCI–H522 cells than in HCT116 cells. The EC_50_ values of PFSE, piceatannol, and scirpusin B for HCT116 were calculated to be 33.7, 96.9, and 92.3 μM, respectively.

**Table 3:** EC_50_ (μM) values as determined by colony formation.

	NCI–H522	HCT116
PFSE	1.92	33.7
Piceatannol	19.0	96.9
Scirpusin	35.5	92.3

EC_50_, half maximal inhibitory concentration. PFSE, passion fruit seed extract.

The WST-8 assay and colony formation experiment revealed that PFSE and its associated stilbenes efficiently inhibited proliferation of NCI–H522 cells, which had higher GLO I expression levels than did HCT116 cells.

## 4 Discussion

Piceatannol, a stilbene abundantly found in passion fruit seeds, reportedly exhibits a variety of biological activities [23]. Recently, we reported that the piceatannol in PFSE prevents high fat diet–induced cardiovascular disease and promotes neural stem cell differentiation to astrocytes [45,46]. In addition, the antiproliferative effects of piceatannol against some cancer cells have been well studied, and several pathways leading to piceatannol-induced apoptosis have been elucidated [21]. For instance, piceatannol suppresses the proliferation of cancer cells by mitochondria-mediated intrinsic pathways, including the PI3K/AKT1/mTOR, spleen tyrosine kinase, cyclooxygenase-2, and IL-6/STAT3 pathways [[25], [26], [27], [28]]. Furthermore, piceatannol has been shown to increased *Fas* and *FasL* mRNA and their corresponding proteins, which extrinsically induce apoptosis in U937 cells [47]. However, it is unknown whether PFSE and stilbenes inhibit proliferation of cancer cells via GLO I inhibition. In the present study, we investigated the effects of PFSE and its stilbene derivatives, piceatannol and scirpusin B, on the cellular proliferation of two types of cancer cells that differ in GLO I expression levels.

Results showed that the stilbenes and PFSE inhibited GLO I enzyme activity (Fig. 1B). PFSE-induced inhibition of GLO I is presumed to result primarily from the action of piceatannol and scirpusin B. As shown in the molecular docking study, in the crystal structure of a human GLO I with baicalein, two hydroxyl groups of baicalein are covalently bonded to zinc ion on human GLO I. As shown in Fig. 2A, piceatannol was predicted to interact with human GLO I, similar to the human GLO I/baicalein complex “binding mode A”. On the other hand, scirpusin B was predicted to be different types of interaction that binding mode A. Thus, one hydroxyl group of scirpusin B is covalently linked to zinc ion on human GLO I (Fig. 2B). We consider that the inhibition effect of GLO I by scirpusin B or piceatannol is affect by the difference binding of zinc ion on GLO I to hydroxyl group of these compounds.

However, the stilbene content and contribution rate indicate that PFSE might contain other components that have an inhibitory affect against GLO I. We also found that NCI–H522 cells had higher expression levels of GLO I protein than did HCT116 cells (Fig. 3A). This is consistent with GLO I gene expression data reported on the CellMiner™ website (https://discover.nci.nih.gov/cellminer/analysis.do). Figs. 3B and 4 show that PFSE and piceatannol inhibits the proliferation of NCI–H522 cells in a dose- and time-dependent manner. Moreover, they reveal that PFSE and piceatannol more significantly inhibited NCI–H522 cell proliferation than HCT116 cell proliferation. Interestingly, PFSE strongly suppressed cancer cell proliferation than piceatannol or scirpusin B in HCT116 and NCI–H522 cells. This finding suggests that the anticancer effects of PFSE are synergistic or additive action of piceatannol, scirpusin B, and other PFSE comprising components (see Table 1). In future, we would like to further investigating the anticancer effects and mechanisms of PFSE in several types of cancer cells.

**Fig. 5:**
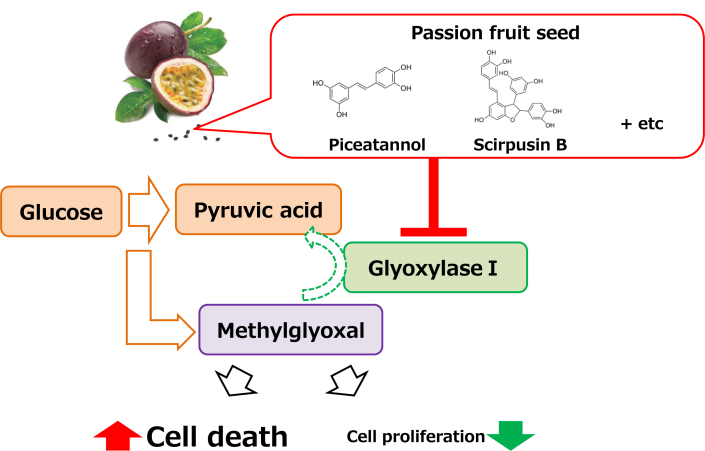
The predicted scheme of anti-cancer mechanism by PFSE.

The above results indicated that the antiproliferative effects of PFSE and piceatannol are GLO I–dependent. Thus, GLO I is a potential target in a novel antitumor pathway, one that might be treated by piceatannol and/or PFSE (Fig. 5). Although piceatannol reportedly binds directly to GLO I [37], overexpression and knockdown of GLO I protein in NCI–H522 and HCT116 cells should be performed to investigate the molecular mechanism of inhibition.

Knowledge of the mechanism of inhibition can aid in investigating potential drug combinations. For example, energy metabolism in cancer cells is dependent upon glycolysis rather than the tricarboxylic acid (TCA) cycle (i.e., the Warburg effect) [5,48]. In cancer cells that predominantly utilize glycolysis, ATP is produced by anaerobic metabolism and MG is produced as the byproduct. To prevent MG-induced apoptosis, cancer cells express GLO I, a detoxification enzyme, to degrade MG. GLO I is the rate-limiting enzyme for detoxification of MG and is highly expressed in cancer cells. When GLO I is inhibited, cancer cells alter energy metabolism, switching from glycolysis to the TCA cycle in order to prevent accumulation of MG and avoid apoptosis [49]. Pyruvate kinase M2 is specifically expressed in many cancer cells and catalyzes the conversion of phosphoenolpyruvate into pyruvate to drive the TCA cycle [50,51]. Shimada et al. reported that the combination of a GLO I inhibitor TLSC702 with shikonin, a pyruvate kinase M2–specific inhibitor, could lead to the suppression of cellular proliferation and induction of apoptosis [52]. Similar combination effects might be expected for PFSE and stilbenes.

In conclusion, while piceatannol has been shown to have possible therapeutic potential against various types of human cancers, this report is the first to investigate the antiproliferative effect of PFSE and associated stilbenes against two types of cancer cells with different expression levels of GLO I. In our study, PFSE and its components, piceatannol and scirpusin B, exhibited beneficial antiproliferative effects in cancer cells. Although *in vivo* and clinical studies will be needed to evaluate the effects of PFSE and stilbene intake, ingestion of PFSE and the stilbenes may aid in the treatment and prevention of cancer via inhibition of GLO I.

## Conflicts of interest

T.Y., M.U., H.M-U., S.M., M.S., and M.M. are employees of Morinaga and Company Limited.

## Author contributions

Designed the study: T.Y., A.S., R.T., S.T. Performed the experiments: T.Y., A.S., Y.T., A.Y., M.U., Y.O., M.I., N.S., A.N., R.I., R.T. Analyzed and discussed the data: T.Y., A.S., R.T., H.M-U., S.M., M.S., M.M., S.T. Wrote the manuscript: T.Y.

## Funding

No funding provided.
